# Multimodal data analysis reveals that pancreatobiliary-type ampullary adenocarcinoma resembles pancreatic adenocarcinoma and differs from cholangiocarcinoma

**DOI:** 10.1186/s12967-022-03473-w

**Published:** 2022-06-15

**Authors:** Jun Cheng, Yize Mao, Wenhui Hong, Wanming Hu, Peng Shu, Kun Huang, Jingjing Yu, Maofen Jiang, Liqin Li, Wei Wang, Dong Ni, Shengping Li

**Affiliations:** 1 National-Regional Key Technology Engineering Laboratory for Medical Ultrasound, Guangdong Key Laboratory for Biomedical Measurements and Ultrasound Imaging, School of Biomedical Engineering, Health Science Center, Shenzhen University, Shenzhen, China; 2 Medical Ultrasound Image Computing (MUSIC) Laboratory, Shenzhen University, Shenzhen, China; 3 Marshall Laboratory of Biomedical Engineering, Shenzhen University, Shenzhen, China; 4 Department of Pancreatobiliary Surgery, Sun Yat-Sen University Cancer Center, Guangzhou, China; 5 Department of Pathology, Sun Yat-Sen University Cancer Center, Guangzhou, China; 6 Molecular Laboratory, Beilun District People’s Hospital, Ningbo, China; 7 Department of Biostatistics and Health Data Science, Indiana University School of Medicine, Indianapolis, IN USA; 8 Regenstrief Institute, Indianapolis, IN USA; 9 Department of Pathology, Ningbo Yinzhou No.2 Hospital, Ningbo, China; 10 Department of Pathology, Beilun District People’s Hospital, Ningbo, China; 11 Huzhou Key Laboratory of Molecular Medicine, Huzhou Central Hospital, Huzhou Hospital Affiliated With Zhejiang University, Huzhou, China; 12 Department of Pathology, The First Affiliated Hospital of Anhui Medical University, Hefei, China

**Keywords:** Ampullary adenocarcinoma, Pancreatobiliary type, Tumor origin, Computational pathology, Adjuvant chemotherapy, Mutation landscape

## Abstract

**Background:**

Ampullary adenocarcinoma (AAC) arises from the ampulla of Vater where the pancreatic duct and bile duct join and empty into the duodenum. It can be classified into intestinal and pancreatobiliary types based on histopathology or immunohistochemistry. However, there are no biomarkers for further classification of pancreatobiliary-type AAC which has important implications for its treatment. We aimed to identify the tumor origin of pancreatobiliary-type AAC by systematically analyzing whole-slide images (WSIs), survival data, and genome sequencing data collected from multiple centers.

**Methods:**

This study involved three experiments. First, we extracted quantitative and highly interpretable features from the tumor region in WSIs and constructed a histologic classifier to differentiate between pancreatic adenocarcinoma (PAC) and cholangiocarcinoma. The histologic classifier was then applied to patients with pancreatobiliary-type AAC to infer the tumor origin. Secondly, we compared the overall survival of patients with pancreatobiliary-type AAC stratified by the adjuvant chemotherapy regimens designed for PAC or cholangiocarcinoma. Finally, we compared the mutation landscape of pancreatobiliary-type AAC with those of PAC and cholangiocarcinoma.

**Results:**

The histologic classifier accurately classified PAC and cholangiocarcinoma in both the internal and external validation sets (AUC > 0.99). All pancreatobiliary-type AACs (n = 45) were classified as PAC. The patients with pancreatobiliary-type AAC receiving regimens designed for PAC showed more favorable overall survival than those receiving regimens designed for cholangiocarcinoma in a multivariable Cox regression (hazard ratio = 7.24, 95% confidence interval: 1.28–40.78, *P* = 0.025). The results of mutation analysis showed that the mutation landscape of AAC was very similar to that of PAC but distinct from that of cholangiocarcinoma.

**Conclusions:**

This multi-center study provides compelling evidence that pancreatobiliary-type AAC resembles PAC instead of cholangiocarcinoma in different aspects, which can guide the treatment selection and clinical trials planning for pancreatobiliary-type AAC.

**Supplementary Information:**

The online version contains supplementary material available at 10.1186/s12967-022-03473-w.

## Background

Ampullary adenocarcinoma (AAC) is a rare malignant neoplasm that arises within the ampullary complex [1], which could originate from three types of epithelial cells: biliary, pancreatic ductal, or duodenal. To date, the suitable chemotherapy regimens for AAC remain in the early exploration stage. The rarity of the disease and complexity of histology were the two main barriers to the exploration of effective chemotherapy regimens for AAC.

AAC can be histologically dichotomized into intestinal and pancreatobiliary types [2]. In most studies, pancreatobiliary-type AAC is found to have worse prognosis than intestinal-type AAC [3, 4]. Cancers that arise from different cellular origins often exhibit different sensitivities to therapeutics [5]. Thus, the chemotherapy regimens for AAC should be different for intestinal and pancreatobiliary types [6, 7]. The recommended regimens for intestinal-type AAC tend to be similar to those for colorectal cancer [6]. However, it is still unclear whether pancreatobiliary-type AAC should be treated like cholangiocarcinoma [8, 9] or pancreatic adenocarcinoma (PAC) [4, 6], as there are no sensitive and specific immunohistochemical markers to determine the tumor origin [10, 11]. Effective techniques to identify the tumor origin of pancreatobiliary-type AAC are in great demand and can greatly promote the development of treatments for this disease in the future.

With the rapid development of sequencing technologies in recent years, researchers have proposed using genomics, transcriptomics, and proteomics to infer tumor origin [10, 12–16]. However, these technologies are not routinely used in clinical practice. Advances in computational pathology have demonstrated great success in classifying cancer types [17–20], predicting cancer prognosis [21–23], and detecting genetic alterations [24–26]. Computational analysis of histopathological images can identify informative and quantitative features that could be too subtle for pathologists to notice.

In this study, we hypothesized that the site of origin of pancreatobiliary-type AAC can be directly inferred from hematoxylin and eosin (H&E) whole-slide images (WSIs). To solve this challenging task, we first developed a WSI-based classification model using the patients with established diagnosis of cholangiocarcinoma and PAC and then used this model to classify patients with pancreatobiliary-type AAC. The classification model was validated using cohorts from multiple sites. In addition, to support the findings of the histopathological analysis, we compared the overall survival of patients with pancreatobiliary-type AAC treated with adjuvant chemotherapies designed for either cholangiocarcinoma or PAC and compared the mutation landscape of pancreatobiliary-type AAC with those of cholangiocarcinoma and PAC.

## Methods

### Study design

**Fig. 1:**
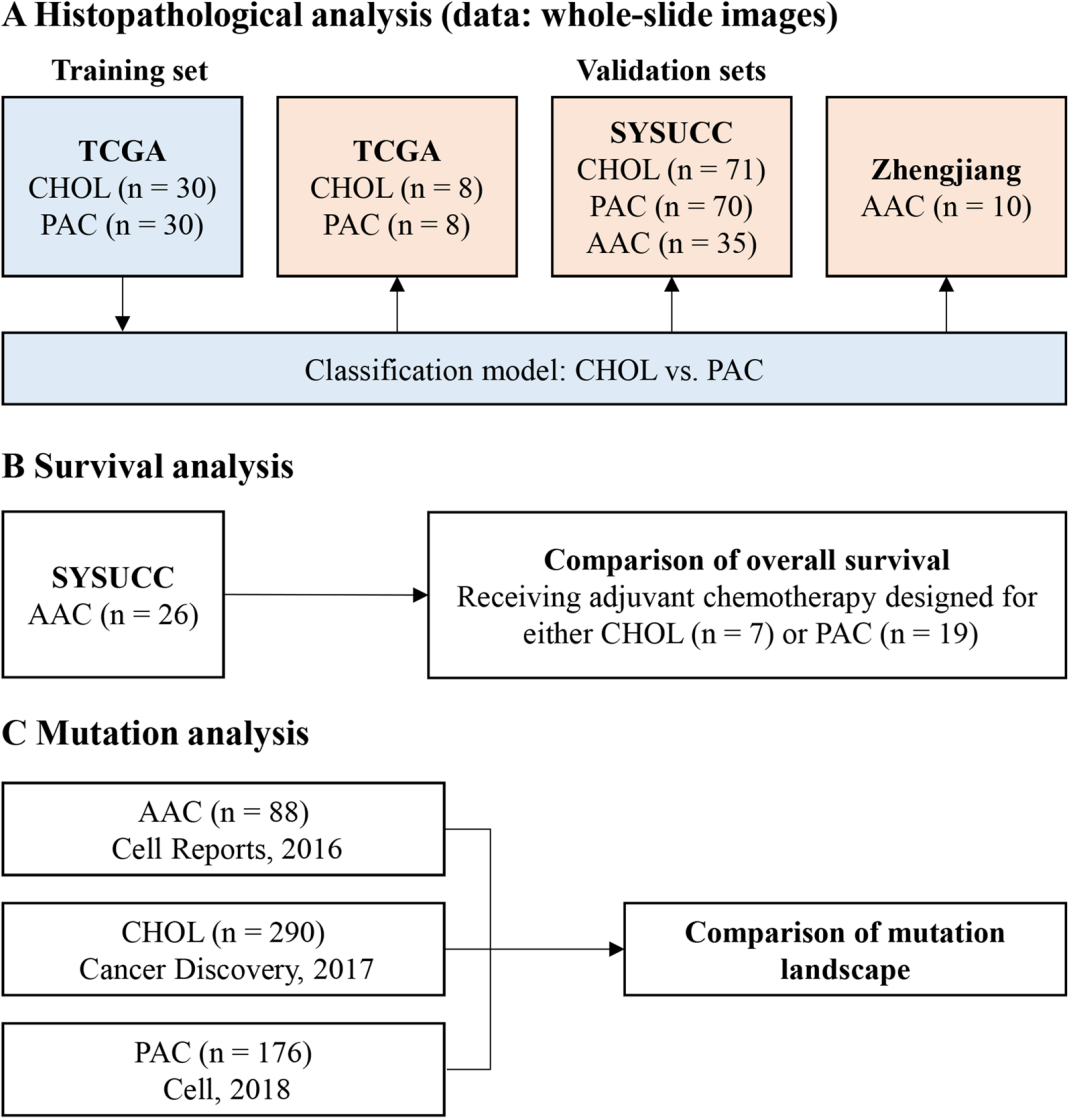
Study design. **A** The classification model of cholangiocarcinoma vs. PAC was trained on the TCGA training dataset. The classification performance was evaluated on the held-out TCGA validation dataset and SYSUCC dataset, and further applied to the patients with pancreatobiliary-type AAC in the SYSUCC dataset and Zhejiang dataset. **B** Overall survival was compared between the pancreatobiliary-type AAC patients who received adjuvant chemotherapy designed for either cholangiocarcinoma or PAC. **C** The mutational landscape of pancreatobiliary-type AAC was compared with those of cholangiocarcinoma and PAC. AAC, ampullary adenocarcinoma; CHOL, cholangiocarcinoma; PAC, pancreatic adenocarcinoma

We performed histopathological analysis, survival analysis, and mutation analysis to investigate whether pancreatobiliary-type AAC is separable. The overview of our study design is shown in Fig. 1. For the histopathological analysis, we constructed a classification model using the H&E WSIs of cholangiocarcinoma and PAC from The Cancer Genome Atlas (TCGA). The model was then validated in the held-out TCGA dataset (internal validation set) and the SYSUCC dataset (external validation set) to evaluate classification performance and applied to the pancreatobiliary-type AACs in the SYSUCC and Zhejiang datasets to infer the tumor origin. For the survival analysis, we investigated whether pancreatobiliary-type AAC patients receiving adjuvant chemotherapy regimens designed for either cholangiocarcinoma or PAC had significantly different overall survival. For the mutation analysis, we explored and compared the mutation landscape of AAC with those of PAC and cholangiocarcinoma, using the genome sequencing data from cBioPortal.

### Patient cohorts

The flowcharts of collecting the WSI datasets and genome sequencing datasets are shown in Additional file 1: Figs. S1, S2, respectively. For the histopathological analysis, three datasets were used. The TCGA dataset consisted of 38 cholangiocarcinoma patients and 38 PAC patients. The SYSUCC dataset consisted of 71 cholangiocarcinoma patients, 70 PAC patients, and 35 pancreatobiliary-type AAC patients from SYSUCC in Guangdong Province, China. The Zhejiang dataset consisted of 10 pancreatobiliary-type AAC patients from Beilun People’s Hospital and Ningbo Yinzhou People’s Hospital in Zhejiang Province, China. The clinicopathological characteristics of the three datasets are summarized in Additional file 1: Tables S1–S3. In these datasets, each patient had one WSI. All WSIs were in SVS format, acquired from formalin-fixed and paraffin-embedded tumor samples after surgical resection, and centrally reviewed by three pathologists (WH, JY, and MJ).

In the TCGA dataset, tissue slides were scanned using an Aperio ScanScope scanner at 40 × magnification (0.25 μm per pixel). Since there are much more PAC cases than cholangiocarcinoma cases in TCGA, we randomly selected 38 PAC cases to match the number of cholangiocarcinoma cases while keeping a similar distribution of tumor stage, tumor grade, and sex. The identifiers of the cholangiocarcinoma and PAC cases used in our TCGA dataset are listed in Additional file 1: Tables S4, S5.

In the SYSUCC and Zhejiang datasets, tissue slides were scanned using a TEKSSQRAY SQS-1000 scanner at 20 × magnification (0.20 μm per pixel). The histopathologic phenotype of AAC was determined based on H&E staining and immunohistochemical staining if necessary. Pancreatobiliary-type AACs are characterized by small solid nest of cells with rounded nuclei surrounded by desmoplastic stroma and forming simple or branching rounded glands. On immunohistochemical staining, they mainly express CK7, CK19, MUC1, MUC5AC, and MUC6. Conversely, Intestinal-type AACs are characterized by tall often pseudostratified columnar epithelium with oval nuclei forming elongated glands. They mainly express CK20, CDX2, SATB2, and MUC2.

For the survival analysis, 26 pancreatobiliary-type AAC patients in the SYSUCC dataset received adjuvant chemotherapy regimens designed for cholangiocarcinoma (n = 7) or PAC (n = 19). The regimens designed for PAC included S-1 based chemotherapy, FOLFIRINOX, gemcitabine plus nab-paclitaxel, or gemcitabine plus capecitabine, while the regimens designed for cholangiocarcinoma included gemcitabine plus oxaliplatin, capecitabine, or gemcitabine plus cisplatin. The clinicopathological characteristics of the patients used for survival analysis are summarized in Additional file 1: Table S6. The overall survival data for these patients were retrieved from clinical records. Overall survival was measured as the time interval between the date of surgery and the date of death or last follow-up. The median and range of follow-up time were 23.3 and 9.8–39.6 months.

For the mutation analysis, we used the genome sequencing datasets for PAC [27], cholangiocarcinoma [28], and AAC [29] from cBioPortal (http://www.cbioportal.org/datasets). After excluding some cases (see Additional file 1: Fig. S2), the resulting 88 pancreatobiliary-type AACs, 290 cholangiocarcinomas, and 176 PACs were used for the mutation analysis. The URLs for these datasets are provided below:

PAC at https://cbioportal-datahub.s3.amazonaws.com/paad_tcga_pan_can_atlas_2018.tar.gz

AAC at https://cbioportal-datahub.s3.amazonaws.com/ampca_bcm_2016.tar.gz

Cholangiocarcinoma at https://cbioportal-datahub.s3.amazonaws.com/chol_icgc_2017.tar.gz

### Computational pathology workflow

**Fig. 2:**
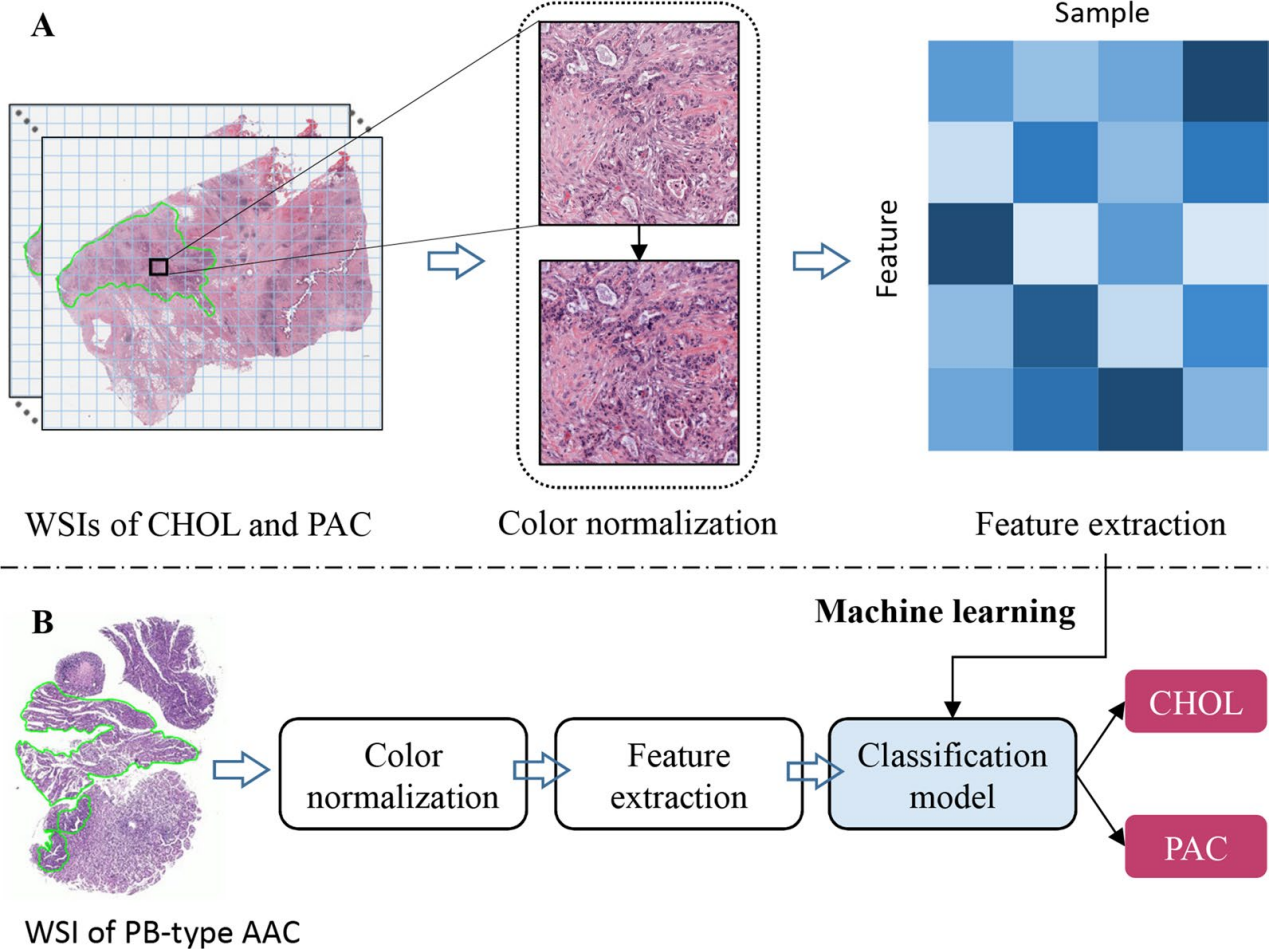
Computational pathology workflow. **A** The classification model to differentiate between cholangiocarcinoma and PAC was built through tumor region annotation in WSIs, color normalization, and feature extraction. **B** Through by similar steps, the features extracted from a WSI of pancreatobiliary-type AAC were input to the classification model to infer the tumor origin. AAC, ampullary adenocarcinoma; CHOL, cholangiocarcinoma; PAC, pancreatic adenocarcinoma; PB, pancreatobiliary; WSI, whole-slide image

Figure 2 shows the computational pathology workflow. We constructed the classification model through manual annotation of tumor region, color normalization, and feature extraction. We then processed the WSIs of pancreatobiliary-type AAC using the same workflow and input the extracted features into the classification model to divide cancers into either cholangiocarcinoma or PAC. The TCGA dataset was divided into the training and validation sets according to a rough ratio of 4:1. We trained a linear SVM classifier based on the top K features selected by the ANOVA F-value using the training set. The two hyperparameters involved in feature selection and model selection, i.e., the number of features K and the regularization parameter C in the linear SVM, were determined by a grid search scheme with five-fold cross validation in the training set. Then, the best hyperparameters were used to train the final classification model using the whole training set, and the model performance was assessed using the untouched validation sets. The linear SVM classifier and feature selection were implemented with a popular machine learning package in python, Scikit-learn v0.19.1.

### Extraction of histopathological image features

The feature extraction process consists of three steps: manual tumor region annotation, color normalization, and feature extraction. The histopathological image features were extracted from the tumor region manually annotated by a pathologist. It is necessary to limit the feature extraction to the tumor region. Otherwise, the classification model built on the WSIs of cholangiocarcinoma and PAC will learn from the features specific to normal pancreas and liver tissue; the normal tissue does not help to identify the origin of tissue of pancreatobiliary-type AAC. The annotated tumor region was cropped into tiles with a size of 2000 × 2000 pixels without overlap to facilitate subsequent analysis. To overcome the undesirable color variations due to, for example, different slide scanners and staining protocols in pathology labs, we used a structure-preserving color normalization algorithm [30] to transform the color appearance of image tiles into that of a target image preferred selected by a pathologist.

We extracted a total of 150 highly interpretable image features for each WSI using the feature extraction algorithm [17] we previously developed. These features quantitatively described the size, staining, shape, and density of cell nuclei. Briefly, for each WSI we first segmented all nuclei and then computed 10 nucleus-level features for each segmented nucleus, including nuclear area (denoted by area); the major axis length, minor axis length, and their ratio (major, minor, and ratio); the mean intensity in R, G, and B channels (rMean, gMean, and bMean); the mean, maximal, and minimal distance to its neighbors (distMean, distMax, and distMin). Finally, we aggregated each type of nucleus-level features into 15 image-level features, including a 10-bin histogram and five distribution statistics (mean, standard deviation, skewness, kurtosis, and entropy). As a result, each image was described by 150 features (10 × 15). Using the nucleus-level feature ratio as an example, the corresponding 15 image-level features were denoted by ratio_bin1 to ratio_bin10, ratio_mean, ratio_std, ratio_skewness, ratio_kurtosis, and ratio_entropy. The histogram features from ratio_bin1 to ratio_bin10 represent the proportions of nuclei with shape varying from round to elongated in the tumor region. More details about the feature extraction pipeline are provided in our previous work [17].

### Statistical analysis

We used a two-sided Mann–Whitney U-test to compare each of the 150 image features between cholangiocarcinoma and PAC. For multiple testing correction, p values were adjusted by the false discovery rate (FDR) procedure according to Benjamini & Hochberg adjustment [31]. We used area under receiver operating characteristic curve (AUC) to assess whether the linear SVM classifier could separate cholangiocarcinoma and PAC. The AUC was computed with the R package pROC v1.15.3. For the pancreatobiliary-type AAC patients who received adjuvant chemotherapy in the SYSUCC dataset, we stratified them into two groups according to the regimens designed for either cholangiocarcinoma or PAC. The Kaplan–Meier method was used to estimate the overall survival, and the log-rank test was performed to test whether the overall survival is significantly different between two groups. A multivariable Cox regression model was used to evaluate the independent prognostic value of the treatment group over sex and histologic grade. For the survival analysis, we used the R package survival v2.43–3. OncoPlot was used to delineate the mutation landscapes of PAC, AAC, and cholangiocarcinoma using the R package maftools v2.6.05. Fisher’s exact test on each gene was performed to detect the differentially mutated genes between two datasets (AAC vs. PAC and AAC vs. cholangiocarcinoma). FDR adjustment was also performed for multiple testing correction. A p value < 0.05 was considered statistically significant, or when FDR adjustment was necessary, an adjusted p value (i.e., q value) < 0.05 was used.

## Results

### Quantitative image analysis identifies significantly different histopathological features between cholangiocarcinoma and PAC

**Fig. 3:**
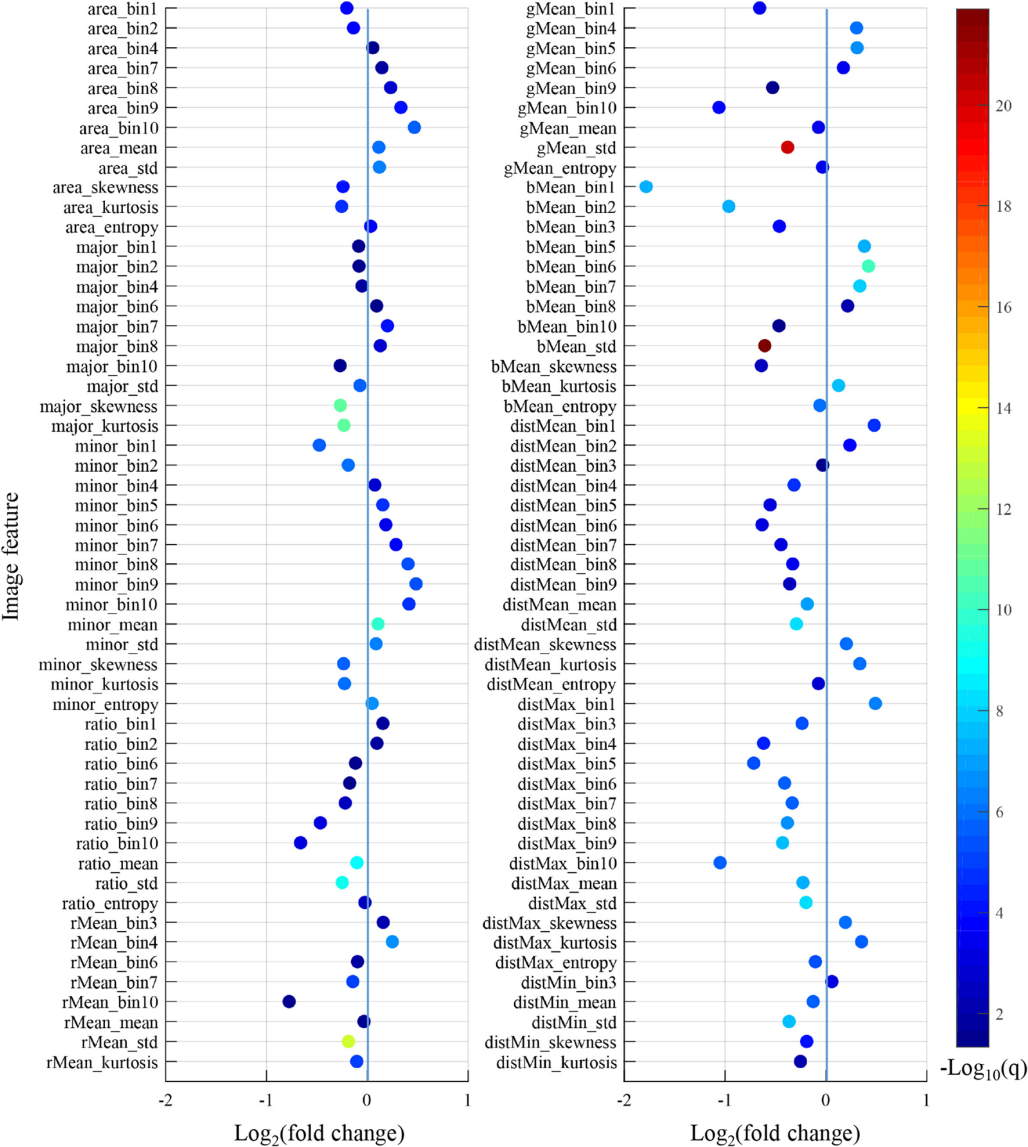
Comparison of image features between cholangiocarcinoma and PAC. Two-sided Mann–Whitney U test was performed for each feature, and 108 out of 150 features showed significant differences between cholangiocarcinoma and PAC after multiple comparison correction using false discovery rate procedure at a 5% level of significance (i.e., q value < 0.05). The fold change for each feature was defined as the ratio of the median feature value between the two groups (cholangiocarcinoma/PAC). CHOL, cholangiocarcinoma; PAC, pancreatic adenocarcinoma

In most cases, pathologists can easily differentiate cholangiocarcinoma and PAC by looking for the normal tissue components in a tissue sample. However, it would be very challenging for pathologists to perform this differentiation by just looking at the tumor region, which is the case for the diagnosis of the tumor origin of pancreatobiliary-type ACC because tumor surrounding tissue now does not provide any useful information. To find the true morphological differences in the two kinds of tumors, we compared each of the 150 quantitative image features extracted from the tumor region using the Mann–Whitney U-test. To enhance the statistical power, we combined the cholangiocarcinoma and PAC samples in the TCGA dataset and the SYSUCC dataset to increase the sample size (n = 109 for cholangiocarcinoma and n = 108 for PAC). Of the 150 image features, 108 features were found significantly associated with the cancer type (q value < 0.05). Figure 3 illustrates the fold change and adjusted p value for each of the 108 features. The fold change for each feature was defined as the ratio of the median feature value between the two groups (cholangiocarcinoma/PAC). A fold change less than 1 means that the median for a specific feature in the cholangiocarcinoma group is less than that in the PAC group.

For the nuclear size related histogram features which describe the proportion of nuclei in a tissue sample from small size to large size, area_bin1 and area_bin2 had fold changes less than one, whereas area_bin4 and area_bin7-10 had fold changes greater than 1. This indicates that cholangiocarcinoma tends to have larger nuclei than PAC. For nuclear shape related features, ratio_bin1 and ratio_bin2 had fold changes greater than one, while the features ratio_bin6-10 had fold changes less than one. The first few bins represent the proportions of relatively round nuclei, and the latter bins represent the proportions of relatively elongated nuclei. Therefore, we can infer that PAC tends to have more elongated nuclei, which essentially are stromal cells, compared with cholangiocarcinoma. Similar analysis of the nucleus density related features such as distMean_bin1-10 and distMean_mean suggests that nuclei are more densely distributed in PAC than cholangiocarcinoma. To the best our knowledge, this is the first study comparing quantitative nuclear features in cholangiocarcinoma and PAC.

### Model predictions reveal that pancreatobiliary-type AAC resembles PAC

**Fig. 4:**
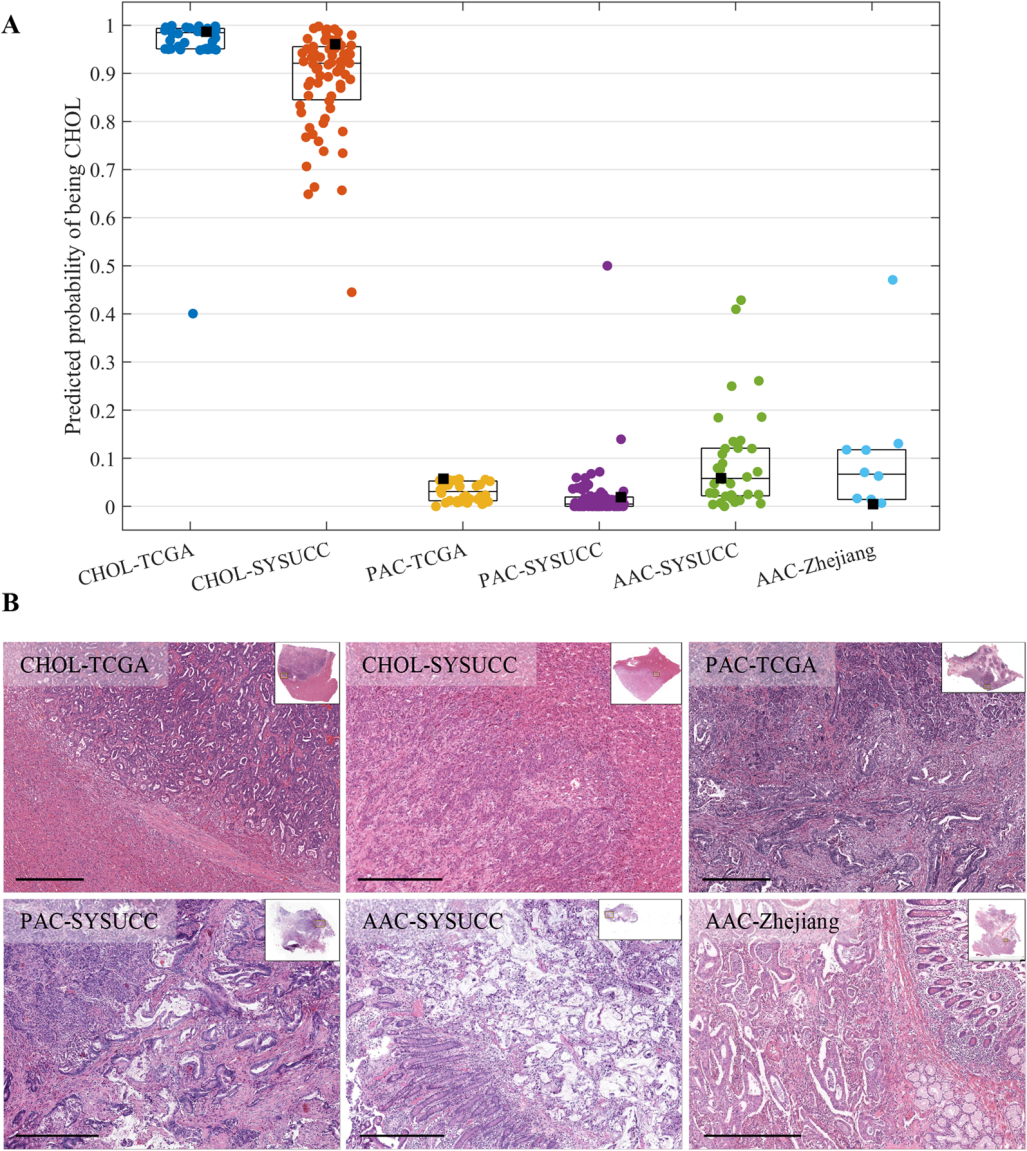
The outputs of the classification model showed that pancreatobiliary-type AAC resembled PAC and differed from cholangiocarcinoma. **A** Scatter plot of the predicted probabilities on patients with cholangiocarcinoma, PAC, and pancreatobiliary-type AAC. The central mark indicates the median, and the bottom and top edges indicate the 25th and 75th percentiles. **B** The corresponding pathological images to the patients indicated by the black squares in the scatter plot. Scale bar: 0.5 mm. AAC, ampullary adenocarcinoma; CHOL, cholangiocarcinoma; PAC, pancreatic adenocarcinoma

We used the patients with an established diagnosis of cholangiocarcinoma or PAC to build a linear SVM classification model to differentiate between the two cancer types. The model was trained with 80% samples of the TCGA dataset and validated using the held-out samples as the internal validation set and the SYSUCC dataset as the external validation set. Additional file 1: Fig. S3 shows the receiver operating characteristic curves for the model in the two validation sets. We can see that the classification model perfectly distinguishes cholangiocarcinoma and PAC (AUC > 0.99 in both validation sets). The model’s outputs for the cholangiocarcinoma and PAC patients in the TCGA and SYSUCC datasets are shown separately in Fig. 4A (model’s outputs for the training samples in the TCGA dataset were also included). We then applied this powerful model to the pancreatobiliary-type AACs in the SYSUCC and Zhejiang datasets. As shown in Fig. 4A, all pancreatobiliary-type AACs were classified as PAC, with the predicted probability of being cholangiocarcinoma less than 0.5. These results indicate that pancreatobiliary-type AAC histologically resembles PAC and differs from cholangiocarcinoma. Figure 4B shows the H&E pathological images of six patients which correspond to the black squares in Fig. 4A.

### Patients with pancreatobiliary-type AAC benefit more from the adjuvant chemotherapy designed for PAC

**Fig. 5:**
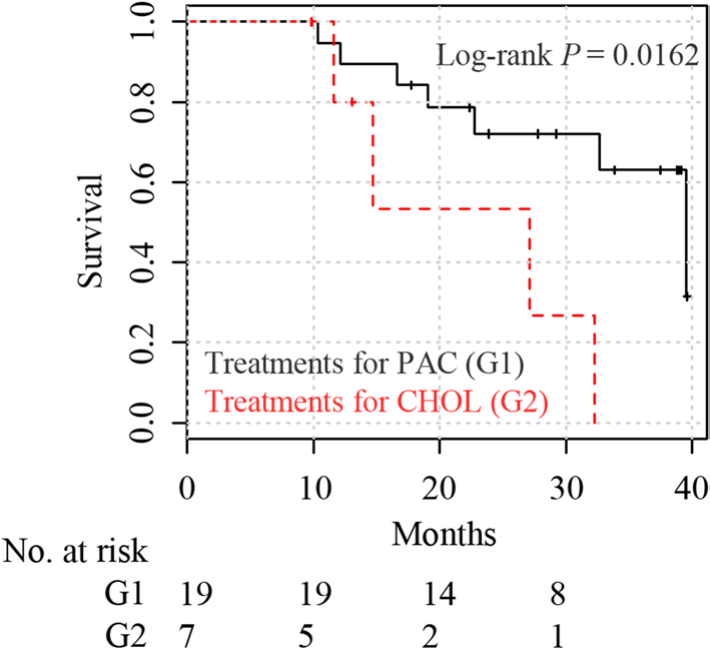
Kaplan–Meier survival curves comparing the overall survival of patients with pancreatobiliary-type AAC receiving adjuvant chemotherapy designed for PAC or cholangiocarcinoma. AAC, ampullary adenocarcinoma; CHOL, cholangiocarcinoma; PAC, pancreatic adenocarcinoma

Now that we have found that pancreatobiliary-type AAC was histologically similar to PAC, we further investigated whether patients with pancreatobiliary-type AAC receiving adjuvant chemotherapy regimens designed for PAC had significantly better prognosis than those treated with regimens designed for cholangiocarcinoma. In the SYSUCC dataset, 19 pancreatobiliary-type AAC patients received the adjuvant chemotherapy designed for PAC while seven received the adjuvant chemotherapy designed for cholangiocarcinoma. Kaplan–Meier survival curves for the two groups are presented in Fig. 5. The one-year and two-year overall survival rates were 94.7% and 72% for the PAC treatment group vs. 80% and 53.3% for the cholangiocarcinoma treatment group. Using Kaplan–Meier estimates, a more favorable overall survival was seen in patients treated with the regimens designed for PAC (log-rank test *P* = 0.0162, Fig. 5). Moreover, the type of adjuvant chemotherapy was significantly associated with survival in a multivariable Cox regression model adjusted for sex and histologic grade (hazard ratio = 7.24, 95% confidence interval: 1.28–40.78, *P* = 0.025). The results of multivariable of Cox regression are shown in Table 1. Although the survival difference between treatment groups is significant, the statistical power may be limited due to the relatively small sample size.

**Table 1:** Results of multivariable Cox regression for overall survival

Variable	HR (95% CI)	*P* value
Adjuvant chemotherapy: for CHOL (vs for PAC)	7.24 (1.28–40.78)	0.025
Sex: male (vs female)	1.32 (0.36–4.76)	0.67
Histologic grade: moderately to poorly (vs moderately)	2.25 (0.43–11.90)	0.34

*CHOL* cholangiocarcinoma, *PAC* pancreatic adenocarcinoma, *HR* hazard ratio, *CI*, confidence interval

### Mutation analysis

**Fig. 6:**
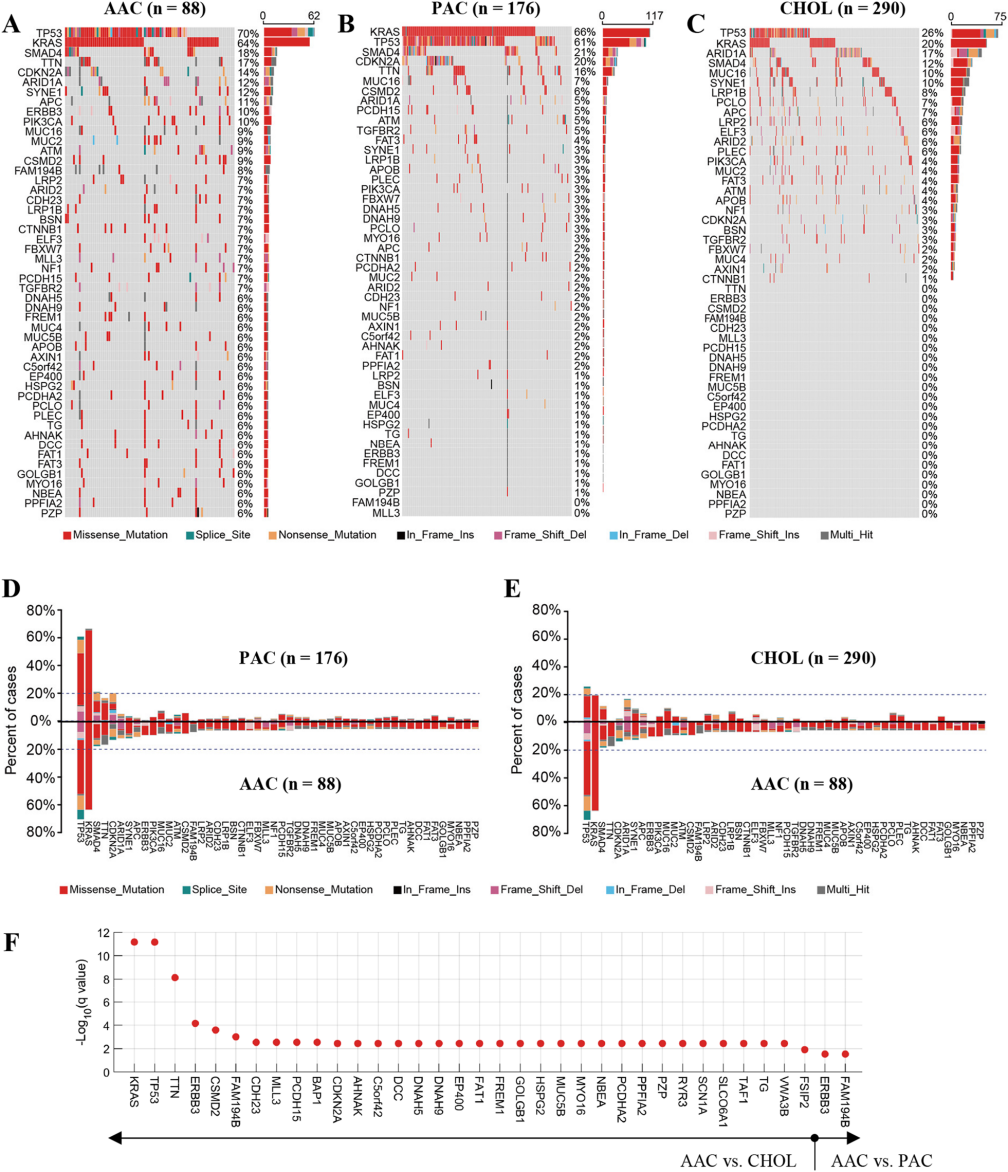
Comparison of the mutational landscape of pancreatobiliary-type AAC with those of PAC and cholangiocarcinoma. **A** Oncoplot summary of the top 50 most frequently mutated genes in pancreatobiliary-type AAC. **B**, **C** oncoplot summary of the same 50 genes in PAC and cholangiocarcinoma. **D**, **E** Paired bar charts of the mutation frequency of the 50 genes for a more intuitive comparison. **F** Fisher’s exact test after multiple test correction identified 34 genes with significantly different mutation frequency between pancreatobiliary-type AAC and cholangiocarcinoma, while only 2 genes were identified between pancreatobiliary-type AAC and PAC. The mutation analysis showed that the mutation landscape of pancreatobiliary-type AAC is similar to that of PAC but different from that of cholangiocarcinoma. AAC, ampullary adenocarcinoma; CHOL, cholangiocarcinoma; PAC, pancreatic adenocarcinoma

Somatic mutation analysis was performed to compare the mutation landscape of pancreatobiliary-type AAC with those of PAC and cholangiocarcinoma. Figure 6A shows the top 50 most frequently mutated genes in pancreatobiliary-type AAC. The mutation rates of this same set of genes in PAC and cholangiocarcinoma are shown in Fig. 6B, C (rows are also rearranged according to mutation prevalence). For a more intuitive comparison between pancreatobiliary-type AAC and the other two cancer types, the paired bar charts of the mutation rate for pancreatobiliary-type AAC vs. PAC and pancreatobiliary-type AAC vs. cholangiocarcinoma are shown in Fig. 6D, E. As we can see, both pancreatobiliary-type AAC and PAC had high frequency of mutations in *TP53* and *KRAS* (more than 60%). We also observed that even though *TP53* and *KRAS* were the most frequently mutated genes in cholangiocarcinoma (Fig. 6C), their mutation rates were only 26% and 20%, respectively, which were much lower than those in pancreatobiliary-type AAC and PAC.

As shown in Fig. 6F, the number of differentially mutated genes between pancreatobiliary-type AAC and PAC was 2, while the number between pancreatobiliary-type AAC and cholangiocarcinoma was 34 (Fisher’s exact test q < 0.05). In addition, we also observed that the characteristically mutated genes in PAC, such as *KRAS* and *TP53*, were the top 2 most differentially mutated genes between pancreatobiliary-type AAC and cholangiocarcinoma. In contrast, the mutation frequency of the two genes were not significantly different between pancreatobiliary-type AAC and PAC. Moreover, previous studies showed that the *CDKN2A* and *TTN* were also the most commonly mutated genes in PAC [32, 33]. The mutation rates of the two genes were not significantly different between pancreatobiliary-type AAC and PAC, whereas they were significantly different between pancreatobiliary-type AAC and cholangiocarcinoma. Together, these data showed the mutation landscape of pancreatobiliary-type AAC was very similar to that of PAC but distinct from that of cholangiocarcinoma.

## Discussion

The difficulty for exploring the regimens for pancreatobiliary-type AAC lies in the lack of reliable techniques to infer the tumor origin. Due to the similar immune-histologic manifestations between pancreatic cancer and biliary cancer, it is difficult to further classify pancreatobiliary-type AAC. Therefore, whether pancreatobiliary-type AAC should be treated like PAC or cholangiocarcinoma is an outstanding issue. To our knowledge, this is the first study that uses computational pathology methods to classify pancreatobiliary-type AAC based on routinely available H&E tissue slides. The experimental results showed that PAC and cholangiocarcinoma are highly distinguishable and that pancreatobiliary-type AAC resembles PAC and differs from cholangiocarcinoma.

Differential diagnosis between PAC and cholangiocarcinoma has significant implications in patient management such as chemotherapy regimens and prognosis but is quite challenging histologically. Both carcinomas present similar histomorphology with infiltrating ductal architecture, mild to moderate nuclear atypia, and dense desmoplastic reaction [34, 35]. Many immunohistochemical markers have been tested to aid pathologists in distinguishing PAC from cholangiocarcinoma [36, 37]. However, because of the overlapping immunohistochemical profiles, most of them are not sensitive and specific enough to be used in clinical practice. In this study, based on ubiquitously available H&E slides, our computational analysis of WSIs identified distinctly different image features between PAC and cholangiocarcinoma. Most importantly, these features are highly interpretable. For instance, we found that cholangiocarcinoma tends to have larger nuclei than PAC and that PAC tends to have denser fibrous tissue than cholangiocarcinoma. These subtle differences cannot be captured by human eyes and have not been reported before.

Previous studies have showed similar clinical outcomes and genomic profiles between pancreatobiliary-type AAC and PAC. Williams et al. [4] reported that pancreatobiliary-type AAC had very similar overall survival to PAC but was significantly more aggressive than intestinal-type AAC. The median overall survival was 33.3, 31.4, and 71.7 months for pancreatobiliary-type AAC, PAC, and intestinal-type AAC, respectively. By genomic sequencing, Yachida et al. [38] showed that the prevalence of driver gene mutations was distinct between pancreatobiliary-type AAC and intestinal-type AAC. Different form previous studies, we, for the first time, systematically compared the mutation landscape of pancreatobiliary-type AAC with those of PAC and cholangiocarcinoma. We found the mutation landscape of pancreatobiliary-type AAC resembled that of PAC but significantly differed from that of cholangiocarcinoma. This indicates that pancreatobiliary-type AAC is likely to originate from the pancreatic duct epithelium instead of the biliary epithelium.

The role of adjuvant therapy for AAC remains controversial [39–41]. For example, Bonet et al. showed that adjuvant therapy after curative-intent resection of AAC was not associated with improved long-term survival [42]. On the other hand, a collaborative study by the Johns Hopkins Hospital and Mayo Clinic reported that adjuvant chemoradiation therapy would improve the outcomes of AAC [43]. There are several reasons for this discordance. First, many studies did not consider the pancreatobiliary-type and intestinal-type separately, which have distinct genomic characteristics and thus should be considered separately. In addition, the regimens of adjuvant therapy used for AAC were varied. The most commonly used chemotherapy regimens for AAC were single gemcitabine; however, gemcitabine alone may be too weak to improve patient outcomes. Ecker et al. reported that gemcitabine-based chemotherapy for pancreatobiliary-type AAC tended to be associated with better survival, though the association was not statistically significant [39]. Since our results showed that all pancreatobiliary-type AACs were classified as PAC, it is worthy to explore whether more effective adjuvant chemotherapy regimens for PAC such as S-1 [44] and mFOLFIRINOX [45] can improve the outcomes of pancreatobiliary-type AAC. Based on our in-house pancreatobiliary-type AAC dataset, we indeed observed that the adjuvant chemotherapy regimens designed for PAC (mainly S-1) was significantly associated with a survival benefit compared with those designed for cholangiocarcinoma (gemcitabine plus oxaliplatin, capecitabine, or gemcitabine plus cisplatin). This supports our argument that pancreatobiliary-type AAC should be considered as and managed like PAC.

This study has several limitations. First, intratumor heterogeneity is unavoidable in studies that take tumor samples and may affect the performance of the histologic classifier. To alleviate the impact of intratumor heterogeneity, we only included the tissue slides obtained from surgical resection specimens which contain a much larger tumor area compared with biopsy specimens. Secondly, due to the rarity of pancreatobiliary-type AAC and only a subset of patients receiving adjuvant therapy, the statistical power may be limited in the survival analysis comparing two treatment groups. Further clinical trials are warranted to validate the effectiveness of applying the adjuvant or first-line chemotherapy regimens for PAC to pancreatobiliary-type AAC.

## Conclusion

This multicenter study provides a promising histologic model for the classification of cholangiocarcinoma vs. PAC and for inferring the tumor origin of pancreatobiliary-type AAC, using routinely available H&E stained diagnostic slides without the extra expense of, for example, genome sequencing. The analyses of different types of data suggest that pancreatic ductal epithelial cells are likely to be the site of origin for pancreatobiliary-type AAC, which could guide clinicians and researchers to select treatments and plan clinical trials for this disease in the near future.

## Supplementary Information

**Additional file 1: Figure S1** Flowchart of collecting the WSI datasets. **Figure S2** Flowchart of collecting the genome sequencing datasets.** Figure S3** Performance of the classification model in classifying cholangiocarcinoma and PAC. **Table S1** Clinicopathological characteristics of the TCGA dataset. **Table S2** Clinicopathological characteristics of the SYSUCC dataset. **Table S3** Clinicopathological characteristics of the Zhejiang dataset. **Table S4** Identifiers of the cholangiocarcinoma cases used in the TCGA dataset. **Table S5** Identifiers of the PAC cases used in the TCGA dataset. **Table S6** Clinicopathological characteristics of the SYSUCC AAC dataset for survival analysis.

## Data Availability

Quantitative features extracted from all H&E stained whole-slide images, survival data, processed genomic sequencing data, and source code are available on GitHub (https://github.com/chengjun583/PB-type-ampullary-adenocarcinoma). The sample identifiers of the whole-slide images used in the TCGA dataset and the download links to the original genomic sequencing data are available in patient cohorts section. Whole-slide images in the SYSUCC and Zhejiang datasets are available from the authors upon reasonable request.

## Abbreviations

AAC: Ampullary adenocarcinoma
AUC: Area under the receiver operating characteristic curve
CHOL: Cholangiocarcinoma
CI: Confidence interval
FDR: False discovery rate
HR: Hazard ratio
H&E: Hematoxylin and eosin
PAC: Pancreatic adenocarcinoma
PB: Pancreatobiliary
ROC: Receiver operating characteristic
SYSUCC: Sun yat-sen university cancer center
TCGA: The cancer genome atlas
WSI: Whole-slide image
