# Supplementary material for: Multimodal data analysis reveals that pancreatobiliary-type ampullary adenocarcinoma resembles pancreatic adenocarcinoma and differs from cholangiocarcinoma
Source: J Transl Med. 2022 Jun 15;20:272. doi: 10.1186/s12967-022-03473-w (PMC9199183; doi:10.1186/s12967-022-03473-w)
Supplement: Supplementary file 1 — Additional file 1: Figure S1 Flowchart of collecting the WSI datasets. Figure S2 Flowchart of collecting the genome sequencing datasets. Figure S3 Performance of the classification model in classifying cholangiocarcinoma and PAC. Table S1 Clinicopathological characteristics of the TCGA dataset. Table S2 Clinicopathological characteristics of the SYSUCC dataset. Table S3 Clinicopathological characteristics of the Zhejiang dataset. Table S4 Identifiers of the cholangiocarcinoma cases used in the TCGA dataset. Table S5 Identifiers of the PAC cases used in the TCGA dataset. Table S6 Clinicopathological characteristics of the SYSUCC AAC dataset for survival analysis. [file 12967_2022_3473_MOESM1_ESM.docx]

**Additional file Information**

**Multimodal data analysis reveals that pancreatobiliary-type ampullary adenocarcinoma resembles pancreatic adenocarcinoma and differs from cholangiocarcinoma**

**Cheng et al.**

**Fig. S1.** Flowchart of collecting the WSI datasets.

**Fig. S2.** Flowchart of collecting the genome sequencing datasets.

**Fig. S3.** Performance of the classification model in classifying cholangiocarcinoma and PAC.

**Table S1.** Clinicopathological characteristics of the TCGA dataset.

**Table S2.** Clinicopathological characteristics of the SYSUCC dataset.

**Table S3.** Clinicopathological characteristics of the Zhejiang dataset.

**Table S4.** Identifiers of the cholangiocarcinoma cases used in the TCGA dataset.

**Table S5.** Identifiers of the PAC cases used in the TCGA dataset.

**Table S6.** Clinicopathological characteristics of the SYSUCC AAC dataset for survival analysis.

# Additional file Figures

**
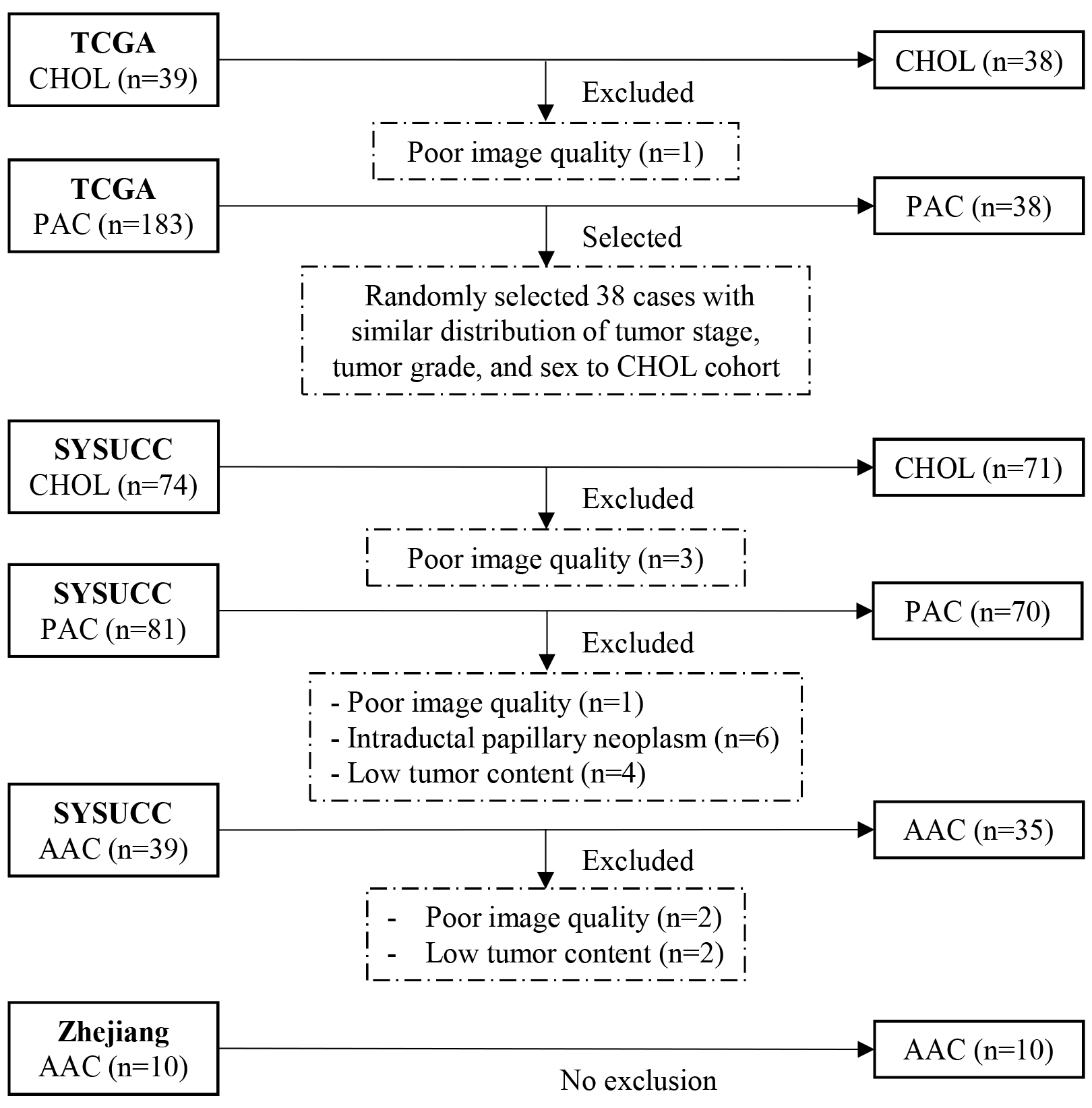
**

**Fig. S1.** Flowchart of collecting the WSI datasets. AAC, ampullary adenocarcinoma; CHOL, cholangiocarcinoma; PAC, pancreatic adenocarcinoma.


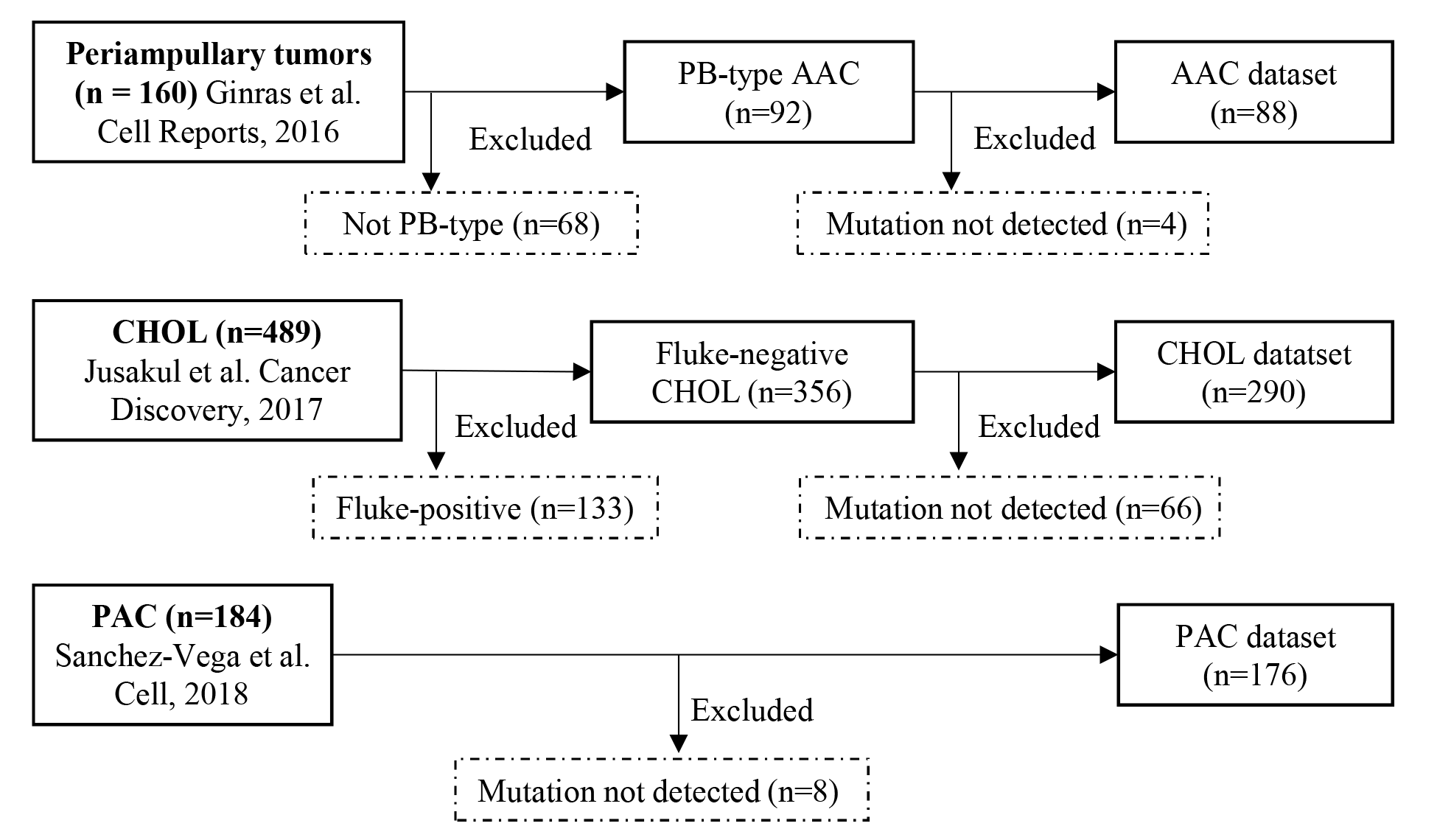


**Fig. S2.** Flowchart of collecting the genome sequencing datasets. AAC, ampullary adenocarcinoma; CHOL, cholangiocarcinoma; PAC, pancreatic adenocarcinoma; PB, pancreatobiliary.


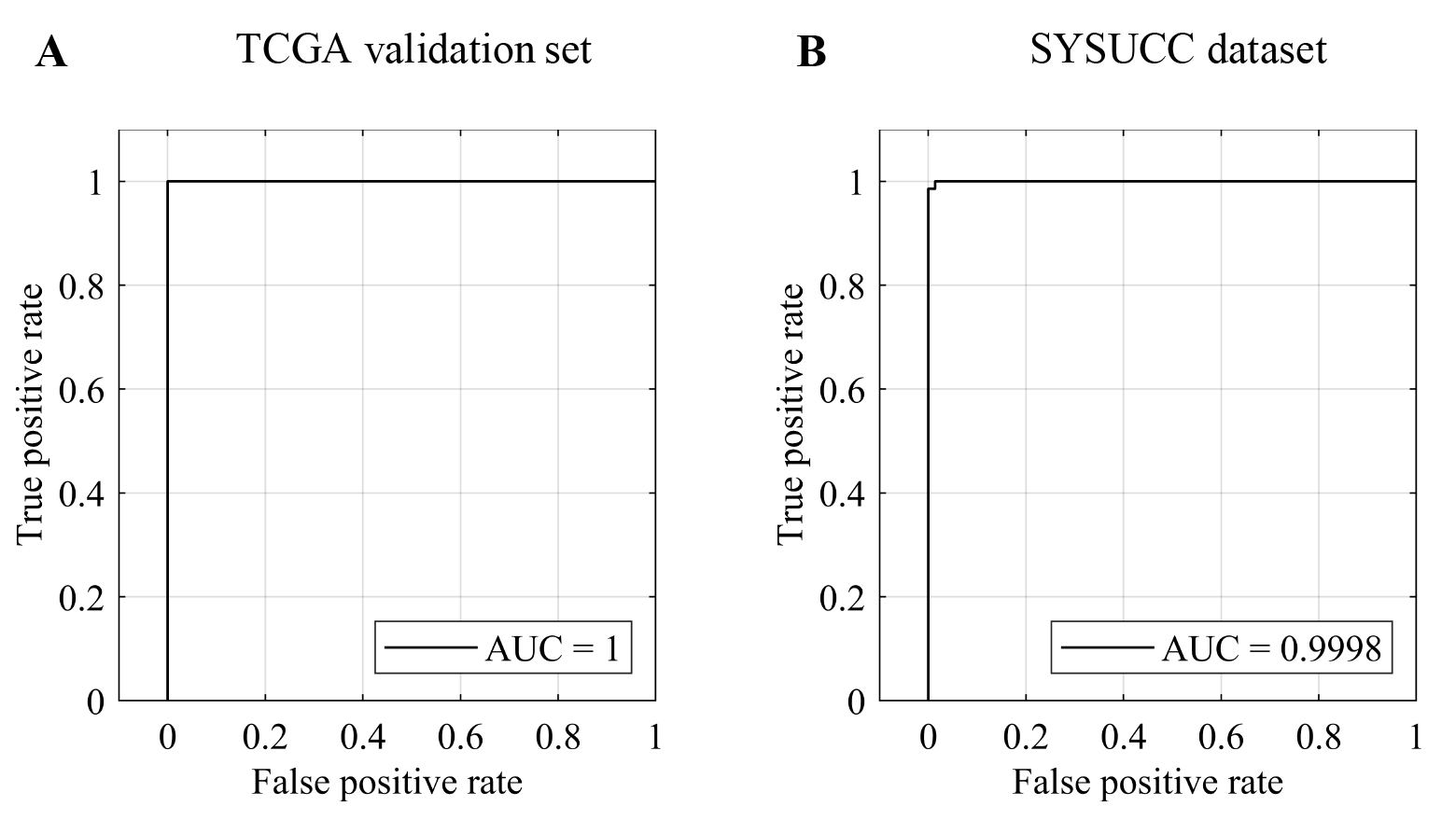


**Fig. S3.** Performance of the classification model in classifying cholangiocarcinoma and PAC in the internal (A) and external (B) validation sets. PAC, pancreatic adenocarcinoma.

# Additional file Tables

**Table S1.** Clinicopathological characteristics of the TCGA dataset. CHOL, cholangiocarcinoma; PAC, pancreatic adenocarcinoma.

| Characteristic | CHOL (n = 38) | PAC (n = 38) |
| --- | --- | --- |
| Age, year |  |  |
| Median | 63 | 64.5 |
| Range | 29-82 | 40-81 |
| Sex, No. (%) |  |  |
| Female | 21 (44.7%) | 19 (50%) |
| Male | 17 (55.3%) | 19 (50%) |
| Histologic grade, No. (%) |  |  |
| G1 | 1 (2.6%) | 1 (2.6%) |
| G2 | 17 (44.7%) | 17 (44.7%) |
| G3 | 18 (47.4%) | 19 (50%) |
| G4 | 2 (5.3%) | 1 (2.6%) |

**Table S2.** Clinicopathological characteristics of the SYSUCC dataset. CHOL, cholangiocarcinoma; PAC, pancreatic adenocarcinoma; PB, pancreatobiliary; AAC, ampullary adenocarcinoma.

| Characteristic | CHOL (n = 71) | PAC (n = 70) | PB-type AAC (n=35) |
| --- | --- | --- | --- |
| Age |  |  |  |
| Median | 52 | 61 | 59 |
| Range | 24-76 | 39-78 | 40-75 |
| Sex |  |  |  |
| Female | 20 (28.2%) | 25 (35.7%) | 12 (34.3%) |
| Male | 51 (71.8%) | 45 (64.3%) | 23 (65.7%) |
| Histologic grade |  |  |  |
| Well | 2 (2.8%) | 1 (1.4%) | 0 |
| Well to moderately | 1 (1.4%) | 2 (2.9%) | 0 |
| Moderately | 19 (26.8%) | 28 (40.0%) | 16 (45.7%) |
| Moderately to poorly | 27 (38.0%) | 32 (45.7%) | 18 (51.4%) |
| Poorly | 22 (31.0%) | 7 (10%) | 1 (2.9%) |

**Table S3.** Clinicopathological characteristics of the Zhejiang dataset. PB, pancreatobiliary; AAC, ampullary adenocarcinoma.

| Characteristic | PB-type AAC (n = 10) |
| --- | --- |
| Age, year |  |
| Median | 66.5 |
| Range | 41-89 |
| Sex, No. (%) |  |
| Female | 6 (60%) |
| Male | 4 (40%) |
| Histologic grade, No. (%) |  |
| Moderately | 2 (20%) |
| Moderately to poorly | 6 (60%) |
| Poorly | 2 (20%) |

**Table S4.** Identifiers of the cholangiocarcinoma cases used in the TCGA dataset.

| Cholangiocarcinoma case ID (n=38) |
| --- |
| TCGA-3X-AAV9-01Z-00-DX1 |
| TCGA-3X-AAVA-01Z-00-DX1 |
| TCGA-3X-AAVB-01Z-00-DX1 |
| TCGA-3X-AAVC-01Z-00-DX1 |
| TCGA-3X-AAVE-01Z-00-DX1 |
| TCGA-4G-AAZO-01Z-00-DX1 |
| TCGA-4G-AAZT-01Z-00-DX1 |
| TCGA-W5-AA2G-01Z-00-DX1 |
| TCGA-W5-AA2H-01Z-00-DX1 |
| TCGA-W5-AA2I-01Z-00-DX1 |
| TCGA-W5-AA2O-01Z-00-DX1 |
| TCGA-W5-AA2Q-01Z-00-DX1 |
| TCGA-W5-AA2R-01Z-00-DX1 |
| TCGA-W5-AA2T-01Z-00-DX1 |
| TCGA-W5-AA2U-01Z-00-DX1 |
| TCGA-W5-AA2W-01Z-00-DX1 |
| TCGA-W5-AA2X-01Z-00-DX1 |
| TCGA-W5-AA2Z-01Z-00-DX1 |
| TCGA-W5-AA30-01Z-00-DX1 |
| TCGA-W5-AA31-01Z-00-DX1 |
| TCGA-W5-AA33-01Z-00-DX1 |
| TCGA-W5-AA34-01Z-00-DX1 |
| TCGA-W5-AA36-01Z-00-DX1 |
| TCGA-W5-AA38-01Z-00-DX1 |
| TCGA-W5-AA39-01Z-00-DX1 |
| TCGA-W6-AA0S-01Z-00-DX1 |
| TCGA-W6-AA0T-01Z-00-DX1 |
| TCGA-WD-A7RX-01Z-00-DX1 |
| TCGA-YR-A95A-01Z-00-DX1 |
| TCGA-ZH-A8Y1-01Z-00-DX1 |
| TCGA-ZH-A8Y2-01Z-00-DX1 |
| TCGA-ZH-A8Y3-01Z-00-DX1 |
| TCGA-ZH-A8Y4-01Z-00-DX1 |
| TCGA-ZH-A8Y5-01Z-00-DX1 |
| TCGA-ZH-A8Y6-01Z-00-DX1 |
| TCGA-ZH-A8Y7-01Z-00-DX1 |
| TCGA-ZH-A8Y8-01Z-00-DX1 |
| TCGA-ZU-A8S4-01Z-00-DX1 |

**Table S5.** Identifiers of the PAC cases used in the TCGA dataset. PAC, pancreatic adenocarcinoma.

| PAC case ID (n=38) |
| --- |
| TCGA-2J-AAB1-01Z-00-DX1 |
| TCGA-2J-AABI-01Z-00-DX1 |
| TCGA-2J-AABR-01Z-00-DX1 |
| TCGA-2J-AABV-01Z-00-DX1 |
| TCGA-3A-A9IB-01Z-00-DX1 |
| TCGA-3A-A9IC-01Z-00-DX1 |
| TCGA-3A-A9IH-01Z-00-DX1 |
| TCGA-3A-A9IN-01Z-00-DX1 |
| TCGA-3A-A9IU-01Z-00-DX1 |
| TCGA-3A-A9IX-01Z-00-DX1 |
| TCGA-3A-A9IZ-01Z-00-DX1 |
| TCGA-F2-6880-01Z-00-DX1 |
| TCGA-F2-7273-01Z-00-DX1 |
| TCGA-FB-A4P5-01Z-00-DX1 |
| TCGA-FB-A5VM-01Z-00-DX1 |
| TCGA-FZ-5920-01Z-00-DX1 |
| TCGA-HZ-8001-01Z-00-DX1 |
| TCGA-HZ-8005-01Z-00-DX1 |
| TCGA-HZ-8519-01Z-00-DX1 |
| TCGA-IB-7644-01Z-00-DX1 |
| TCGA-IB-7887-01Z-00-DX1 |
| TCGA-IB-7890-01Z-00-DX1 |
| TCGA-IB-7893-01Z-00-DX1 |
| TCGA-IB-A5SQ-01Z-00-DX1 |
| TCGA-IB-A7M4-01Z-00-DX1 |
| TCGA-IB-AAUN-01Z-00-DX1 |
| TCGA-IB-AAUO-01Z-00-DX1 |
| TCGA-IB-AAUQ-01Z-00-DX1 |
| TCGA-L1-A7W4-01Z-00-DX1 |
| TCGA-L1-A7W4-01Z-00-DX4 |
| TCGA-L1-A7W4-01Z-00-DX5 |
| TCGA-LB-A9Q5-01Z-00-DX1 |
| TCGA-PZ-A5RE-01Z-00-DX1 |
| TCGA-RB-A7B8-01Z-00-DX1 |
| TCGA-RL-AAAS-01Z-00-DX1 |
| TCGA-US-A77E-01Z-00-DX1 |
| TCGA-XD-AAUG-01Z-00-DX1 |
| TCGA-XD-AAUG-01Z-00-DX2 |
| TCGA-XD-AAUG-01Z-00-DX3 |
| TCGA-XD-AAUG-01Z-00-DX4 |
| TCGA-XD-AAUL-01Z-00-DX1 |
| TCGA-XN-A8T3-01Z-00-DX1 |
| TCGA-YY-A8LH-01Z-00-DX1 |

**Table S6.** Clinicopathological characteristics of the SYSUCC AAC dataset for survival analysis. PB, pancreatobiliary; AAC, ampullary adenocarcinoma.

| Characteristic | PB-type AAC (n = 26) |
| --- | --- |
| Age, year |  |
| Median | 59.5 |
| Range | 33-75 |
| Sex, No. (%) |  |
| Female | 10 (38.5%) |
| Male | 16 (61.5%) |
| Histologic grade, No. (%) |  |
| Moderately | 10 (38.5%) |
| Moderately to poorly | 16 (61.5%) |
| Adjuvant chemotherapy |  |
| Designed for PAC | 19 (73.1%) |
| Designed for chol | 7 (26.9%) |
| Follow-up (month) |  |
| Median | 23.3 |
| Range | 9.8-39.6 |
| Death No. (%) | 11 (42.3%) |
